# Wideband and high-order microwave vortex-beam launcher based on spoof surface plasmon polaritons

**DOI:** 10.1038/s41598-021-02749-3

**Published:** 2021-12-02

**Authors:** Lei Zhang, Min Deng, Weiwen Li, Guang Yang, Longfang Ye

**Affiliations:** 1 Department of Electronic Engineering, Xiamen University, Xiamen, 361005 China; 2 Red Phase Inc., Xiamen, 361008 China; 3 Institute of Electromagnetics and Acoustics, Xiamen University, Xiamen, 361005 China; 4 State Key Laboratory of Millimeter Waves, Southeast University, Nanjing, 210096 China

**Keywords:** Engineering, Electrical and electronic engineering

## Abstract

The electromagnetic vortex carrying orbital angular momentum (OAM), which is first studied at optical frequency, has begun to attract widespread attention in the field of radio-frequency/microwave. However, for the OAM mode generated by traditional single antennas, there are problems such as low order and narrow bandwidth, and complex structures such as dual-fed networks may be required. In this paper, based on spoof surface plasmon polariton (SSPP) mode leaky-wave antenna, a single-port traveling-wave ring is proposed to radiate high-order OAM modes working near the cut-off frequency of SSPP state. The achieved 12-order OAM mode within 9.1–10.1 GHz (relative bandwidth of 10.4%) has the main radiation direction close to the antenna surface, forming a plane spiral OAM (PSOAM) wave, which reduces the requirements for mode purity in practical applications. This SSPP ring using periodic units as radiating elements can be an effective radiator for broadband and large-capacity OAM multiplexing communications. The structural characteristics of single feed contribute to the integration of microwave circuits.

Field components of the vortex wave carrying orbital angular momentum (OAM) have Hilbert phase term $e^{ - jl\varphi }$, where the integer $l$ presents OAM topological charges and $\varphi$ is azimuth angle^1^. Since OAM modes are confirmed at optical frequencies, OAM waves have been widely used in super-resolution imaging^2–4^, quantum information technologies^5–7^, optical communications^8^. At radio-frequency (RF)/microwave bands, OAM modes have also been used to improve channel capacity and spectrum utilization of wireless communications^9,10^. In this regard, the generation of OAM modes can be divided into passive and active method. After a plane wave passes through a spiral phase plate, an additional Hilbert phase is introduced to achieve passive vortex generation. The phase factor can also be obtained by a reflecting paraboloid with spiral phase. Of course, this kind of phase plate or reflective surface can be replaced by holographic plates or metasurfaces, to form a rich variety of phase generation structures for realizing OAM modes. However, these passive devices will inevitably introduce insertion loss, and the OAM order obtained is fixed. The ideal ultimate method should be to directly generate the OAM mode from the radiation source, such as optical vortex beam emitters^11^, without the need for an intermediate mode conversion process. We call this the active vortex generation.

In microwave frequency band, the use of a circular phased array is the simplest and most direct method of active generation of OAM waves. As *N* array elements are placed at equal intervals along the circumference, and each element is excited with equal amplitude and phase shift $\Delta \phi$ of $l \cdot 2\uppi = N \cdot \Delta \phi$, *l*-order OAM beam is generated^12^. Nevertheless, a complicated feed network with delay lines is essential in this structure. On the other hand, for the circular time-switched array antenna, high-speed RF switches are required^13^. For this reason, the use of simple single antennas to directly radiate OAM modes, including loop antennas, patch antennas, and dielectric resonant antennas, has attracted more and more attention^14–16^.

Theoretically, the higher the OAM topological charges are, the more the multiplexing channels and the larger the information capacity for transmission. However, the OAM mode order generated by a single antenna is generally low, and the mode purity decreases significantly as the order increases. For patch antennas, a third-order OAM mode can be obtained with the help of the characteristic mode synthesis method^14^. Using the whispering gallery mode, a third-order OAM dielectric antenna has also been implemented^15^. At present, ring traveling-wave currents mainly radiate OAM mode within 5 orders^16^. Although a method to generate OAM modes is theoretically feasible, it is not necessarily the case in actual implementation, especially for the realization of higher-order modes. The distribution fineness of the phase and amplitude of OAM beams is related to the wavelength. For this reason, few OAM modes with more than 10 orders are achieved in the microwave frequency band. Meanwhile, the generation of higher-order OAM requires a large radiation structure and a complex feed network. For boresight OAM beams, on the other hand, as the order increases the divergence angle increases and the phase singularity range expands, which is unfavorable for longer distance communication. Fortunately, plane spiral orbital angular momentum (PSOAM), which main propagation is in the radial direction close to antenna surface, has been suggested to provide a new solution for the application problem of high-order OAM beam divergence^17,18^.

It has been conceived to use a circular leaky-wave antenna to achieve OAM mode^19^. Since its OAM mode realization is essentially based on a traveling wave loop, a large structure is also required to obtain a higher-order mode. In this case, using a leaky wave antenna based on spoof surface plasmon polariton (SSPP) mode is an effective way to reduce the structure size. SSPP mode operating at microwave frequencies is a kind of slow wave similar to optical surface plasmon polaritons, which has the characteristics of near-field enhancement, surface confinement, and deep subwavelength^20–24^. Since SSPP modes propagate only along the metal/dielectric interface with periodic slots or holes and the ground plane is omitted, the corresponding devices can also be thinned. In fact, lower-order OAM modes using ring SSPP waveguide have been achieved^25–27^. However, the radiator is a dual-port structure, which causes part of the energy to be directly transmitted to another port and also increases the size of entire structure^26^, or double-sided processing is required, thereby introducing discontinuous areas and increasing manufacturing complexity^25^. There has been a ring OAM antenna based on single-port fed SSPP structure, but the radiation is generated by coupling elements and the structure size is still large^27^. Through the analysis of the radiation and transmission characteristics of SSPP leaky wave structures, we have proposed the concept of single-port SSPP leaky wave antennas^28^. In this paper, single-fed leaky-wave emitters based on SSPP mode are proposed to radiate high-order, broadband, and near-plane OAM waves. As a specific example, an OAM antenna based on H-shaped periodic units realizes 12-order planar spiral orbital angular momentum in 9.1–10.1 GHz. In this regard, the feature of the single-port SSPP is firstly analyzed and then the high order OAM launcher is constructed. Finally, the OAM mode performance, including the frequency characteristics of topological charge and radiation pattern, is measured and discussed.

## Results

### Initial single-port SSPP antenna

**Figure 1:**
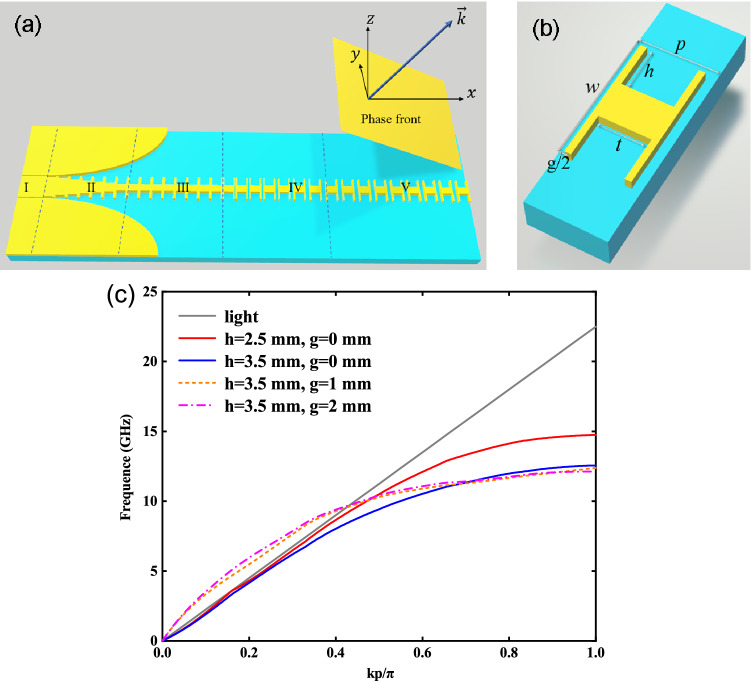
(**a**) Structural illustration of the initial single-port leaky wave antenna based on H-shaped periodic units, which is composed of five areas. (**b**) Structural parameters of the corresponding H-shaped periodic unit. (**c**) Dispersion characteristics of H-shaped periodic units.

There are three main methods for SSPP waveguide to achieve leaky wave radiation, i.e. introducing cutting gaps into the waveguide^29^, sinusoidally modulating its shape^30^, and periodically loading coupling elements^31^. Essentially, they are all based on the modulation of surface wave impedance. For the leaky wave antenna based on a slotted SSPP waveguide, the bound fields after only a few periodic cells may be very weak. In this case, a single port structure can be used to realize the leaky wave antenna function. To effectively radiate OAM waves, the initial single-port leaky wave antenna with H-shaped periodic units as shown in Fig. 1a is first constructed and analyzed. The enlarged view for the periodic unit is presented in Fig. 1b.

The initial antenna prototype is fabricated by using F4BK microwave substrate with a permittivity of 2.2, loss tangent of 0.01, and thickness of 0.8 mm. For the H-shaped periodic cell, its period is *p* = 5.0 mm, the conductor width is *w* = 10.0 mm, the groove depth is *h* = 3.5 mm, and the groove width is fixed at *t* = 4.0 mm. The cut slit width on both sides of an H-shaped cell is *g*/2. Using the eigenmode analysis method, the obtained dispersion characteristics of periodic units are shown in Fig. 1c. While *g* = 0.0 mm, that is, there is no cut gaps for SSPP waveguide, the dispersion curve as a whole is on the right side of light cone, showing a strongly bound SSPP transmission mode. In this case, as the groove depth *h* decreases, the dispersion curve moves closer to the optical cone. Thus, adjustment of groove depth can be used to perform the momentum transition between the traditional transmission line and SSPP waveguide, which corresponds to part II of the single-port antenna as shown in Fig. 1a. With the introduction of cut gaps, the dispersion curve is divided into a fast wave area that is good for radiation, a slow wave area that is good for transmission, and a cut-off frequency area. The change of the slit width *g* can plays an impedance matching transition in the fast wave region, so part IV is introduced in the single-port leaky wave antenna. In order to enhance the conversion from TEM mode to SSPP mode, part III is also introduced in the initial antenna.

Therefore, the single-port antenna as shown in Fig. 1a includes five structural areas. Part I is a coplanar waveguide (CPW) as the feed port with a central conductor strip of width 10.0 mm, two slits of width 0.5 mm, and two ground planes of width 25.0 mm and length 10.0 mm. Parts II and III are the mode transition and momentum matching areas. In order to simultaneously achieve impedance matching, the shape of two ground planes is 1/4 ellipse with a semi-major axis of 50.0 mm and semi-minor axis of 25.0 mm. The center conductor in part II is composed of 7 periodic units, but the groove depth *h* is uniformly increased from 0.5 mm to 3.5 mm. The conductor strip of part III is composed of 8 H-shaped units with groove depth *h* = 3.5 mm, which functions as a SSPP transmission line. There are 8 structural units in part IV, but the gap *g* between adjacent units uniformly increases from 0.25 mm to 2.0 mm. 8 periodic units with slit width of *g* = 2.0 mm are arranged in part V to realize effective leakage wave radiation.

The detailed performance of initial leaky-wave antenna prototype is presented in the supplementary material. Its frequency characteristics are consistent with the analysis results obtained from the dispersion curves. The effective and sufficient radiation can be achieved by adopting a smaller number of periodic units in part V, confirming that single port leaky wave antenna is reliable. We can also infer that by adjusting the structural parameters of periodic units, especially the slit width *g*, and then controlling the bound ability of SSPP modes, the length of the effective radiation area can be regulated. That is, the number of periodic units that need to generate effective radiation can be controlled, thereby providing a construction basis for the realization of subsequent high-order OAM mode antennas.

For a SSPP leaky-wave antenna, the angle between the maximum radiation and normal direction (+ *z* axis) of the antenna surface, i.e. radiation angle $\theta_{r}$, can be written as^32^

1 $$ \theta_{r} = \arcsin \left[ {\frac{1}{{k_{0} }}\left( {\frac{d\phi }{{dx}} + k_{spp} } \right)} \right], $$

where $$k_{0}$$ is the free space wave number, $${\raise0.7ex\hbox{${d\phi }$} \!\mathord{\left/ {\vphantom {{d\phi } {dx}}}\right.\kern-\nulldelimiterspace} \!\lower0.7ex\hbox{${dx}$}}$$ is the phase gradient of a periodic unit for SSPP waveguide, and $$k_{spp}$$ is the wave number of SSPP mode. Since the phase gradient and SSPP wave number increase with frequency, the main radiation surface is inclined to the *xoy* plane where the antenna is located with the increase of working frequency. This is also verified by the radiation field characteristics of initial antenna, as shown in Supplementary Fig. S7 of the Supplementary Information. Therefore, in order to generate PSOAM radiation mode, the operating band should be as close as possible to the cutoff frequency of SSPP waves.

### High order OAM launcher structure

If the radiation region of initial single port leaky wave antenna is coiled into a ring, the traveling wave loop current which can radiate OAM mode will be formed. To realize high-order OAM modes, the number of radiation units should be increased in the tail of single port SSPP leaky wave antenna. Since the path length, i.e. ring perimeter *C*, should be an integral multiple of the corresponding waveguide wavelength for the ring traveling wave current, the specific condition for the generation of *l* order OAM vortex waves in a ring structure is

2 $$ C = \left| l \right|\frac{{2\uppi }}{{k_{spp} }}, $$

where the positive and negative of *l*, i.e. the direction of vortex wave, is determined by the direction of traveling wave. According to $k_{spp}$, the corresponding waveguide wavelength *λ*_*g*_ can be calculated.

According to the basic structure parameters of initial single port antenna, an OAM mode radiation structure of annular traveling wave is constructed. The cut-off frequency of SSPP mode transmission line for initial single port antenna is around 12.5 GHz, while the intersect frequency point between the dispersion curve of the radiation units and the light cone is about 10.0 GHz. Therefore, 9.5 GHz is chosen as the design frequency of the loop OAM antenna. On the one hand, this frequency is helpful for PSOAM mode realization. On the other hand, due to the relatively strong binding ability, SSPP modes can be distributed in long radiation region, thereby achieving high-order OAM modes. According to the dispersion curves of the radiation units in Fig. 1c, the wave vector at this frequency is $k_{spp} = 188.4{\kern 1pt} \,\text{rad/m}$, and the waveguide wavelength is $\lambda_{g} = 33.3\,\text{mm}$. If the traveling wave ring is designed according to 12-order OAM mode, the length of the ring structure should be about 400 mm and the ring radius should be about 63 mm.

**Figure 2:**
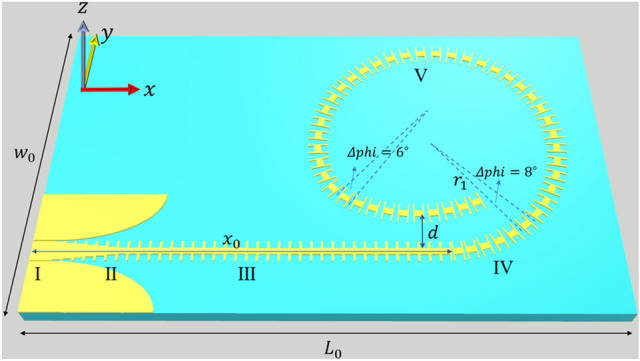
High-order OAM single-port antenna structure based on H-shaped periodic units.

The final high-order OAM mode loop antenna is shown in Fig. 2, which is manufactured on microwave substrate with $L_{0}$ = 270.0 mm and $W_{0}$ = 160.0 mm. Similar to the initial leaky wave antenna, it also consists of five parts. The structure and size of part I and II are exactly the same as the initial antenna. However, in this case the quarter elliptical ground planes are all included in part II. Part III is completely an SSPP slow wave transmission line. In order to reduce the influence of CPW grounding planes on the ring current, the unit number in part III is increased to 30.

Part IV is the transition region from SSPP transmission unit to radiation unit. In order to avoid excessive radiation in + *x* direction, this part is taken as the starting point of traveling wave loop. In this way, the SSPP units with cut gaps rotate anticlockwise with a radius of $r_{1}$ = 60.0 mm from − *y* axis. In addition, for the six periodic cells in part IV, the corresponding center angle $\Delta phi$ starts from 5.5° and increases uniformly to 8° in a step of 0.5°.

Part V is the main radiation region of OAM mode, which is divided into two parts. The first part is made up of 31 periodic units rotating in a radius of $r_{1}$ = 60.0 mm and a fixed center angle of 8° for each unit, immediately after part IV. The second part is a total of 10 periodic cells starting from the 32nd radiating unit, which are still circling with the same radius. However, in order to avoid strong coupling with the SSPP transmission mode in part III, the rotation center for this arc is moved upward in *y*-axis direction with a distance of *d* = 20.0 mm. On the other hand, to maintain strong coupling fields within this radiation section, the central angle of periodic cells is reduced to 6°. In this case, there are three more periodic cells in the radiation ring region than that of a single closed ring, so the total traveling wave path length is approximately $2\uppi r_{1} + 3p$ = 398 mm, which agrees well with the estimated value.

### OAM launcher performance

**Figure 3:**
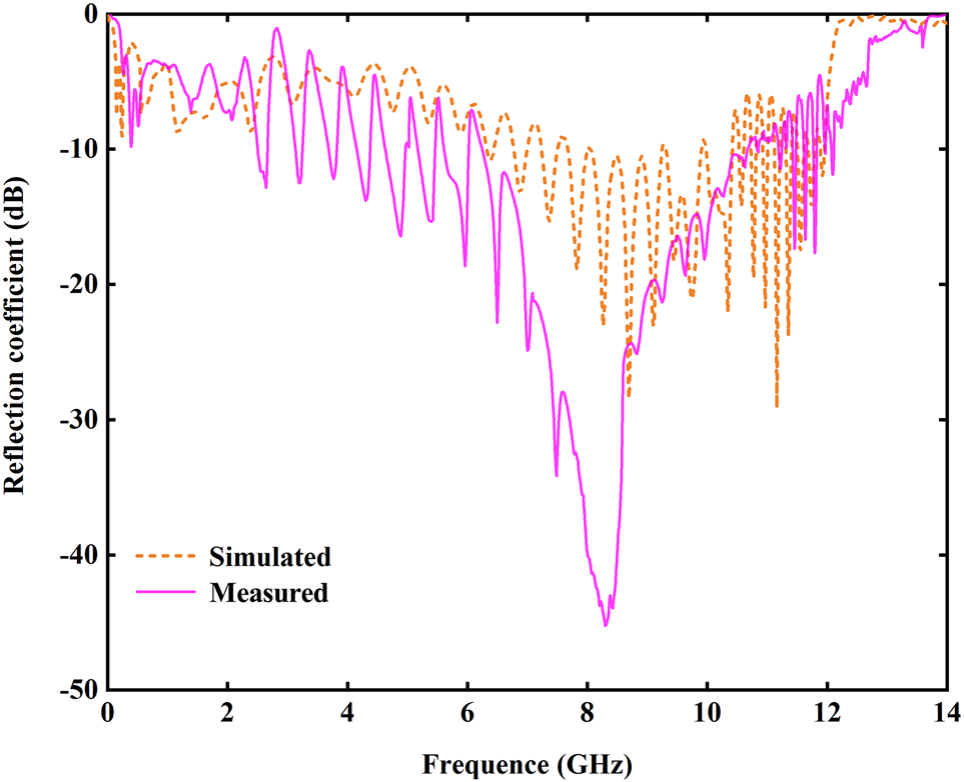
Impedance characteristics of high-order OAM mode single-port loop antenna.

The port scattering characteristics of OAM antenna prototype shown in Fig. 3 confirms that the variation trend for the simulation results is consistent with the measured. However, with reflection coefficient *S*_11_ less than − 10 dB, the test band is 6.5–11.0 GHz (the relative bandwidth of about 66%), and the simulation range is 8.0–10.4 GHz (~ 26%). The difference between the two is caused by the machining error and substrate loss. Since the simulated band range is within the measured, and the band should be close to the cut-off frequency to generate near-plane radiation, this simulation band is used as the discussing frequency range. According to the simulation results, the cut-off frequency at high-frequency end is around 12.3 GHz for this antenna, close to the asymptotic frequency of 12.5 GHz corresponding to the dispersion curve.

Since the unit period *p* has a relatively gentle effect on SSPP mode dispersion^33^ and as shown in Fig. 1c the gap spacing between the periodic cells has also little influence on it, the OAM order in the discussed band is estimated by using Eq. (2), and the results are listed in Table 1. It can be seen that the antenna has the similar OAM order in a larger frequency range. This corresponds to the larger slope of dispersion curves on the right side of light cone shown in Fig. 1c.

**Table 1:** Theoretical parameter values of OAM mode at different frequencies.

*f* (GHz)	*k*_*spp*_ (rad/m)	λ_*g*_ (mm)	OAM order *l*
9.0	178.3	35.2	11.3
9.1	183.9	34.1	11.7
9.6	191.5	32.8	12.2
10.1	197.4	31.8	12.5
10.2	201.8	31.1	12.8

**Figure 4:**
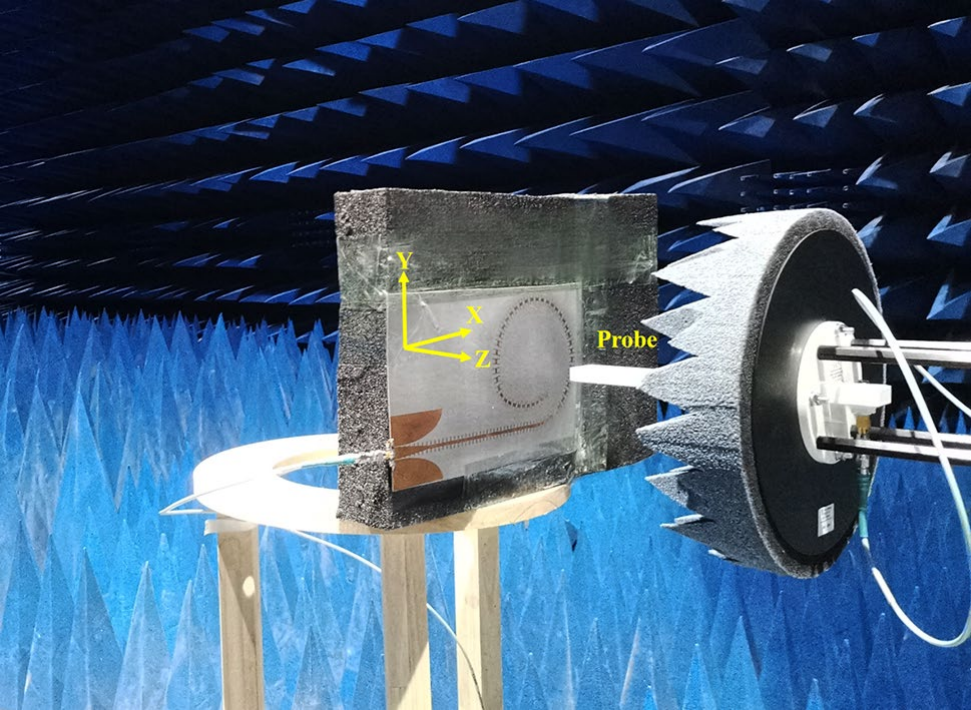
Experimental platform in an anechoic chamber for antenna performance test.

**Figure 5:**
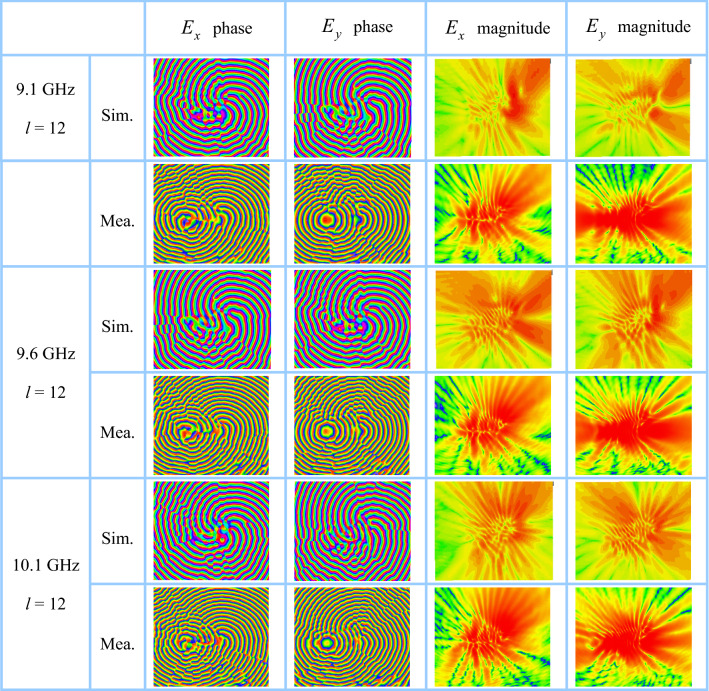
The phase and magnitude distributions of electric field components *E*_*x*_ and *E*_*y*_ on a 800 mm × 800 mm plane with 55 mm away from the antenna surface.

Combining port impedance analysis and OAM order estimation of the proposed antenna, the near-field electric fields at 9.1, 9.6, and 10.1 GHz are tested by using NSI-MI antenna measurement system in a microwave anechoic chamber, as shown in Fig. 4. The test and simulation results on the rectangular observation plane with a size of 800 mm × 800 mm at *z* = 55 mm (1.8 $\lambda$@ 9.5 GHz) from the antenna surface are listed in Fig. 5. According to experimental results, a 12-order OAM mode is generated in the range of 9.1–10.1 GHz. As the frequency is lower than 9.1 GHz, the OAM mode can still be generated. However, the order will decrease due to the increase of waveguide wavelength. On the contrary, as the frequency is higher than 10.1 GHz, 13th order mode should be produced. Certainly, the phase distribution distortion of higher-order OAM modes is serious. The OAM orders shown in Fig. 5 are consistent with the results listed in Table 1.

As shown in Fig. 5, there approximately are two phase singularities for each field component near the observation plane center. The reason is that the travelling-wave ring is composed of two parts that do not share the same center. The equivalent loop is elongated in the lateral direction. Due to the non-concentric superposition of the left and right fields, large field strength is also distributed at the geometric center where the OAM fields should be hollow. The phase lines of OAM mode are distorted to a certain extent. Coupled with the influence of feed port, the field phase distributions near the feedline side are more uneven. Relatively, the phase in the upper right half is stable, so the near-plane OAM mode in this direction can be better utilized in practical applications.

**Figure 6:**
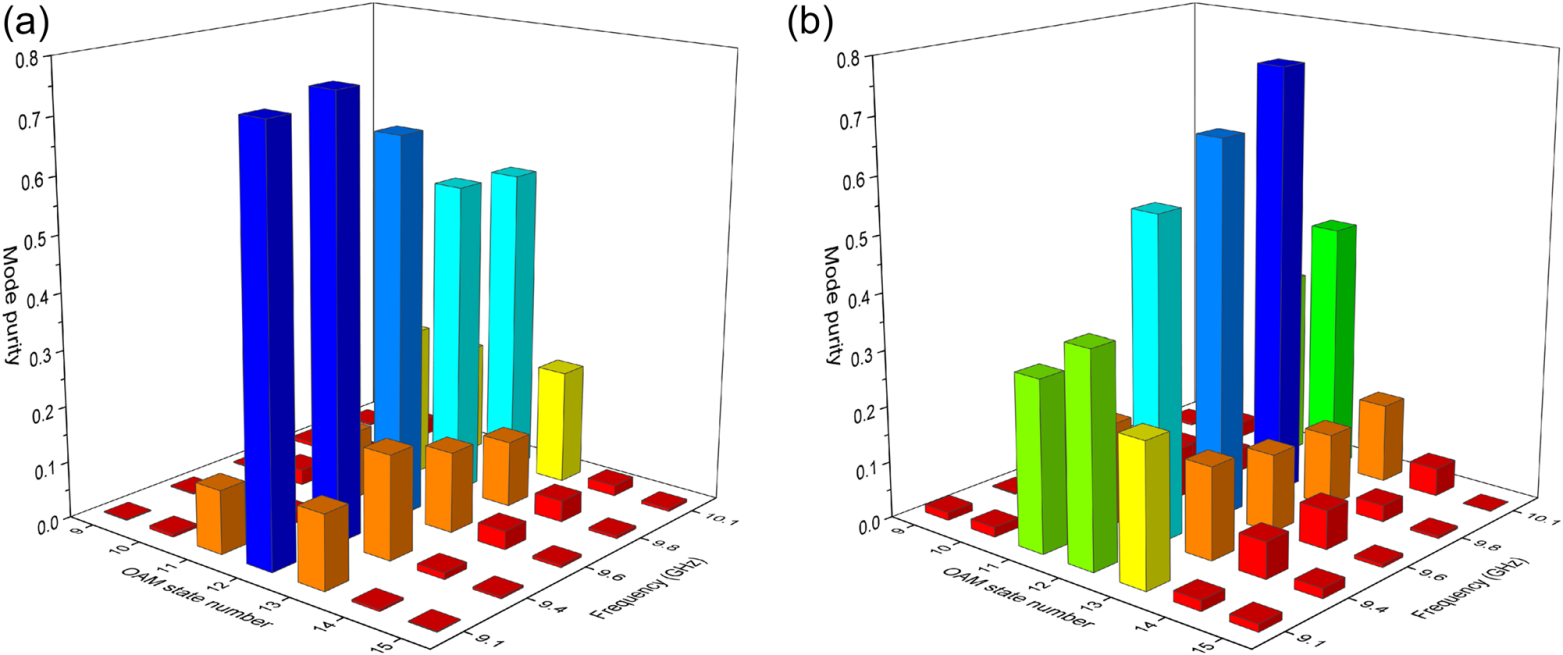
The normalized mode purity of the beam emitted from the proposed OAM antenna. (**a**) The modal content calculated through simulated phase data. (**b**) The modal content calculated through measured phase data.

By using Fourier transform to the mode fields^34,35^, the mode index weights as shown in Fig. 6 are obtained. It can be seen that in the range of 9.1–10.1 GHz, the main modes are all 12-order OAM waves. So this frequency range can be the final actual working zone. However, due to the asymmetry of the ring structure and the influence of the feedline, the mode purity is a bit low.

**Figure 7:**
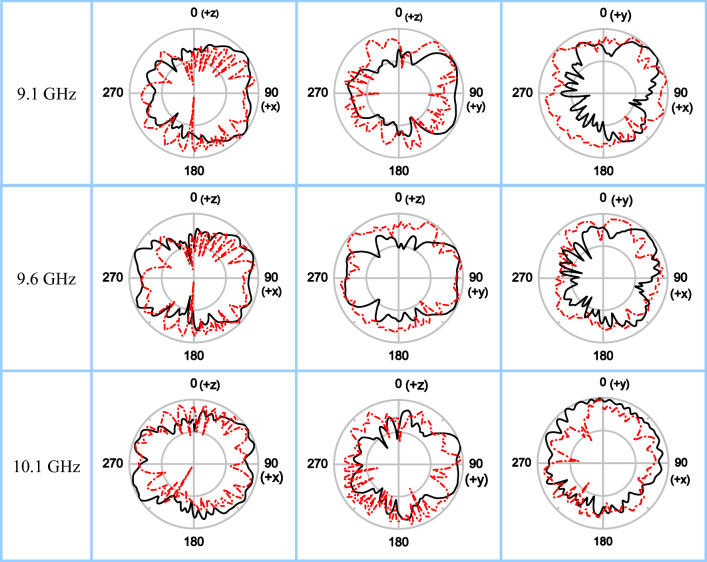
Normalized radiation patterns in *xoz*-, *yoz*-, and *xoy*-plane at 9.1, 9.6, and 10.1 GHz (black solid line for simulated results and red dash dot line for measured results).

The antenna radiation patterns are also tested in the MSI-MI system. The measured and simulated patterns in *xoz*-, *yoz*- and *xoy*-cut planes at 9.1, 9.6, and 10.1 GHz are shown in Fig. 7. It can be seen that there is an approximate minimum value of far-field strength in the normal direction of antenna surface (*z* axis direction, with theta = 0°), which is the vortex wave's singularity in boresight direction. According to the simulation results, as the frequency increases from 9.1 GHz to 10.1 GHz, the maximum radiation direction in *xoz*-plane gradually varies from theta = 54° to 66°, and in *yoz*-plane the radiation theta angle increases from 60° to 70°, all approaching the antenna surface. The measured results have similar trends. In *xoy*-plane, due to the feedline influence, the maximum radiation direction is more tilted towards the area between + *x* and + *y* axes. That is, the strongest radiation is around phi = 45°. It can be seen from Fig. 5 that the mode resolution in this direction is also high, so this part of OAM mode fields should be fully utilized in practical applications of PSOAM. Using the radial radiation of PSOAM can solve the problem of receiving OAM mode caused by large field hole in the boresight direction for traditional high-order OAM beam. It can also eliminate the adverse effects of uneven OAM phase distribution on signal reception, and thus the requirements for mode purity are reduced.

## Discussion

Based on SSPP mode leaky wave radiation, a single-port traveling wave loop can be constructed to generate a high-order PSOAM with planar radiation. In this paper, a SSPP antenna with a stable 12-order OAM mode in 9.1–10.1 GHz has been achieved. Its main radiation is close to the radial direction of antenna plane. That is, PSOAM vortex waves are generated, which reduces the requirements for mode purity.

This single-port antenna can also greatly reduce the device size. Obviously, near the cutoff frequency, the proposed antenna realizes the extremely high-order OAM modes with a wide band. In Ref.^25^, using a SSPP-based ring structure with a radius of 80.0 mm, the second-order OAM mode is achieved near 6.0 GHz, and it is sensitive to frequency. Even if the frequency factor is considered, the structure size of our proposed antenna is greatly reduced, which contributes to the device miniaturization.

The SSPP structure in Refs.^25,27^ just functions as a transmission line, and the circular coupling patches produce the radiation effect similar to the antenna array elements. For the proposed antenna, the SSPP structure itself is also an OAM mode radiator. Of course, due to the dual-port structure and the lower order, the OAM mode purity in Refs.^25,27^ is high. For this proposed antenna, the symmetry of the ring structure and the feed method should be considered comprehensively to improve the mode purity.

Although the mode order can be regulated by controlling the operating frequency, a more effective and direct method is to adjust the coupling between periodic units. This kind of broadband, high-order, and miniaturized antennas can be used for large-capacity omni-directional communications, multi-directional detection, and so on. And this implementation method can be applied to the generation of OAM modes in millimeter wave and terahertz frequency bands.

## Methods

The simulation analysis is carried out by using the electromagnetic software CST Studio Suite. The corresponding dispersion characteristic analysis uses its eigenmode solver, while the reflection coefficients, near field distributions, and the radiation patterns use the Time Domain solver.

The actual structures of the antennas are manufactured by the surface mask etching method. After the substrate surface is masked, an etching solution composed of ferric chloride and water is used to etch the copper layer. Then the reflection coefficients of the fabricated emitters are measured by using an vector network analyzer AV3629D.

The near-field phase and magnitude distributions are measured by using NSI-MI near-field antenna measurement system in an anechoic chamber. In this case, a horn antenna is used as a probe to measure the electric field components $E_{x}$ and $E_{y}$. The antenna radiation patterns are also tested in this MSI-MI system. After the components $E_{x}$ and $E_{y}$ of far-field electric fields in three orthogonal planes have been measured, the total field patterns are synthesized for these planes.

## Supplementary Information

Supplementary Information.
