# Supplementary material for: Wideband and high-order microwave vortex-beam launcher based on spoof surface plasmon polaritons
Source: Sci Rep. 2021 Dec 2;11:23272. doi: 10.1038/s41598-021-02749-3 (PMC8639978; doi:10.1038/s41598-021-02749-3)
Supplement: Supplementary file 1 — Supplementary Information. [file 41598_2021_2749_MOESM1_ESM.doc]

**Supplementary Material**

**Wideband and High-Order Microwave Vortex-Beam Launcher Based on Spoof Surface Plasmon Polaritons**

Lei Zhang, Min Deng^2^, Weiwen Li^*^, Guang Yang^1^, and Longfang Ye^3,4^

*Department of Electronic Engineering, Xiamen University, Xiamen 361005, China*

^2^ *Red Phase Inc., Xiamen 361008, China*

^3^*Institute of Electromagnetics and Acoustics, Xiamen University, Xiamen 361005, China*

^4^*State Key Laboratory of Millimeter Waves, Southeast University, Nanjing 210096, China*

*E-mail: *wwl@xmu.edu.cn*

**1. Scattering characteristics of initial single-port leaky wave antenna**

According to the construction size of initial single-port leaky wave antenna in the main text, its prototype (as shown in Fig. S1) was made by etching method, and the impedance performance was tested. Figure S2 shows the simulation and test results, which are in good agreement. Obviously, this single-port leaky wave antenna has broadband impedance characteristics. The measured frequency band of S_11_ less than – 10 dB is 5.2–9.5 GHz, the center frequency is 7.9 GHz, and the relative bandwidth reaches 54.3%.


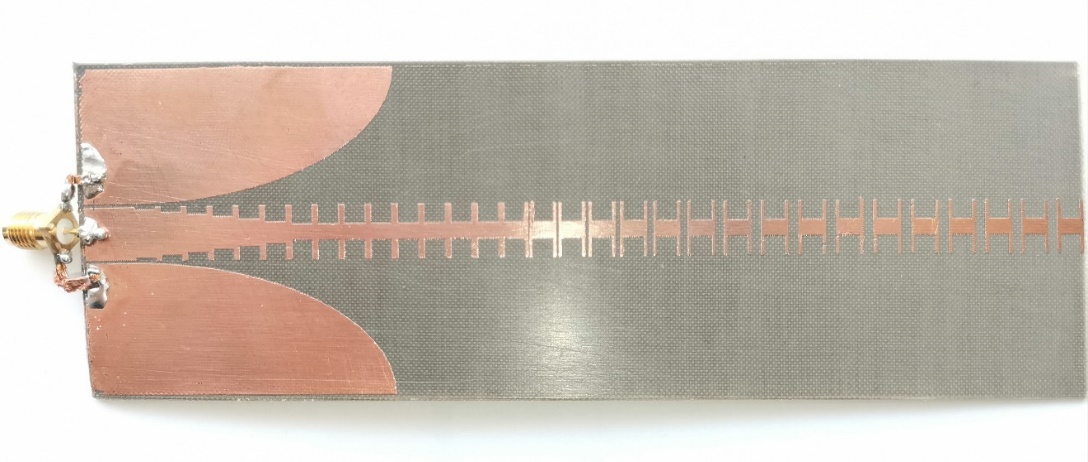


**FIG. S1.** Photograph of initial single-port leaky-wave antenna prototype based on SSPP modes with H-shaped periodic units. See the main text for specific structural parameters, dielectric substrate and test conditions.


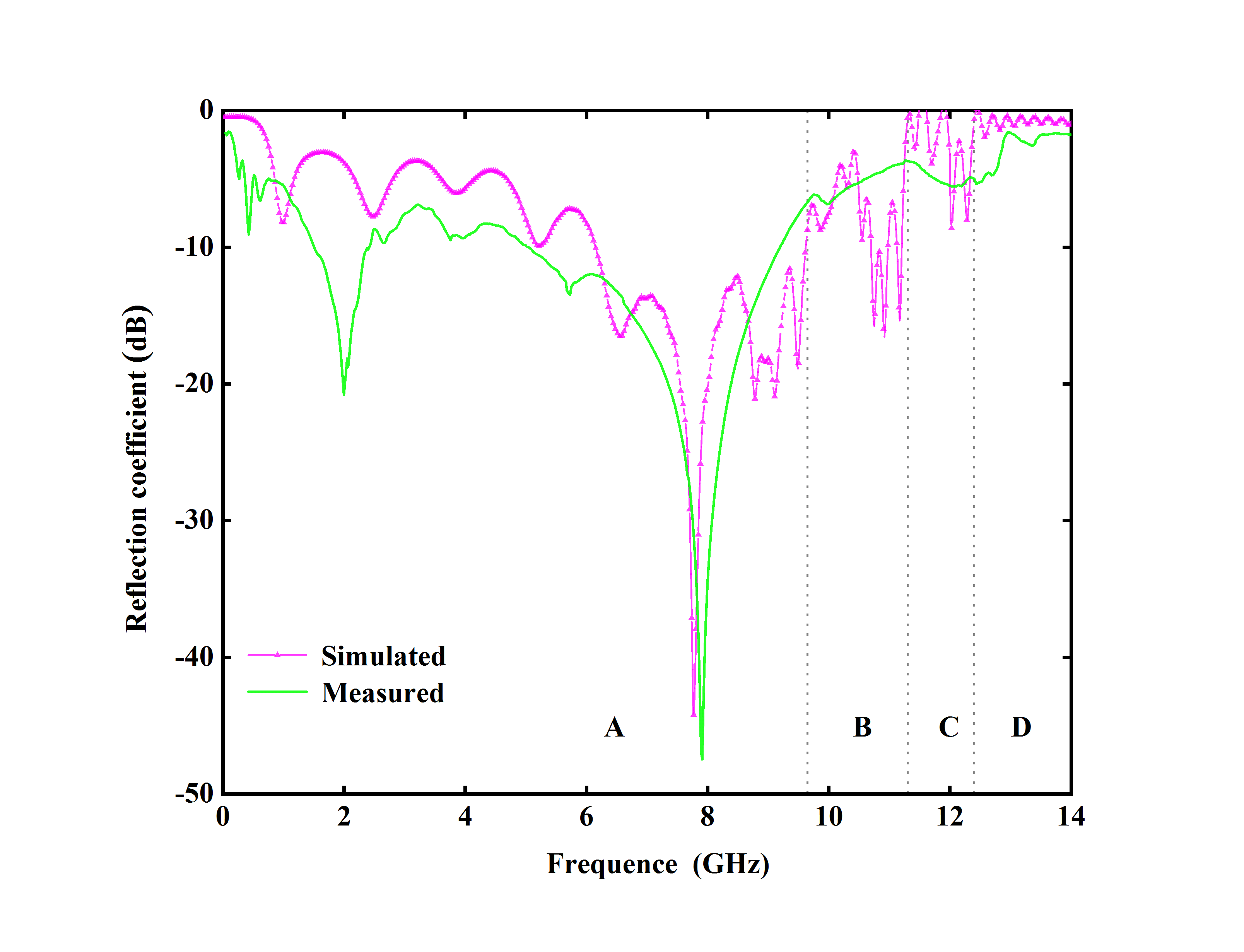


**FIG. S2.** Impedance characteristics of initial single-port leaky wave antenna. In order to correspond to the analysis results from the dispersion curves of periodic units, four regions A, B, C, and D are marked in the figure.


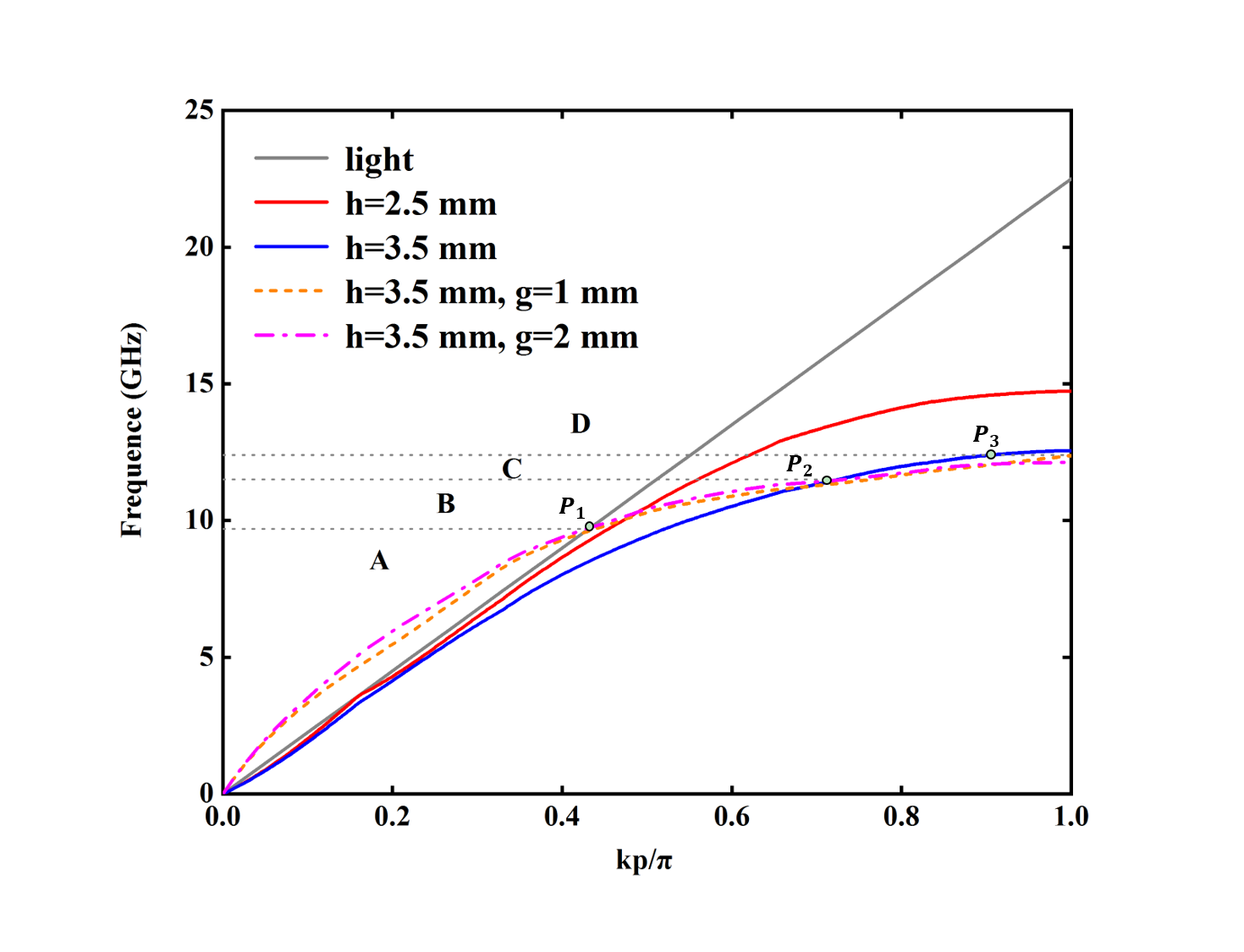


**FIG. S3.** Dispersion characteristics of H-shaped periodic structure, corresponding to Fig. 1(c) in the main text. The structural parameters of H-shaped periodic units can be found in the main text. The straight line in the figure represents the light cone. The solid line on the right side of light cone corresponds to the dispersion as there are no cut gaps between periodic cells. In this case the slow wave propagation mode with strong confinement appears, and the asymptotic frequency drops significantly as the groove depth *h* increases. While cutting gaps are introduced between the periodic units, dispersion curves are located on the left side of light cone at low frequency end, showing fast-wave radiation characteristics. Combining the dispersion characteristics of the two periodic structures, it can be divided into four frequency regions. The frequencies below the intersection point *p*_1_ of the dispersion curve for the slotted periodic structure and the light cone line correspond to zone A. The point *p*_2_ is the intersection of two dispersion curves for the periodic structures with and without cutting gaps. The frequencies from point *p*_1_ to *p*_2_ are in zone B. From point *p*_2_ frequency to the point *p*_3_, the asymptotic frequency of the slotted periodic structure, we have zone C. The frequencies above point *p*_3_ are in zone D.

The frequency characteristics of the port scattering shown in Fig. S2 can correspond to the dispersion effect of periodic structures shown in Fig. S3, and thus mutual verification analysis is performed.

SSPP mode transmission line is a slow-wave structure, which has strong bound ability to fields, and its dispersion curve is located on the right side of light cone. But for the periodic structure introduced with the cutting gaps, the dispersion curve at low frequency end, the range of 0.0–10.0 GHz as shown in Fig. S3, moves to the left of light cone curve, showing fast-wave characteristics. And SSPP leakage radiation is generated. Therefore, for the initial single-port antenna, good port matching is achieved below about 10.0 GHz.

In the band of 10.0–11.5 GHz, i.e. the B region of Fig. S3, the dispersion curve for the slotted periodic cells is located below the light cone and above the dispersion curve of the periodic units without gaps. At this time, the radiation mode of SSPPs is converted to slow mode. The slotted periodic structure in this frequency region has strong restraint ability to fields, and the radiation effect is weakened. Meanwhile, in the cut-off area of SSPP waveguide, the SSPP mode transmission is deteriorated. Therefore, in the B zone of port impedance performance, the port matching is significantly worse.

For area C in Fig. S3, the curves of slotted periodic units are below that for the SSPP waveguide without gaps. Although SSPP modes can be introduced to the slotted units by SSPP waveguide, the bound ability in this slotted area is further increased, and the radiation effect is further weakened. Corresponding to the port scattering, the matching performance is further deteriorated, which is the characteristic of the C area in Fig. S2.

The frequencies in the D area of Fig. S3 have entered the cut-off area of the SSPP waveguide, so effective radiation cannot be realized. Corresponding to S_11_ parameter, it is in a state of total reflection.

Obviously, the frequency regions of dispersion curve for the periodic structures can well correspond to the S_11_ characteristics of the initial single-port antenna. Thus, our analysis and design for this single-port antenna are reasonable. It also shows that as a leaky wave antenna, the application band should be lower than the frequency at point *p*_1_, that is, below about 10.0 GHz.

**2. Determination of radiation area length**

This leaky wave antenna realizes radiation by introducing cutting slots into the SSPP waveguide. If a dual-port structure is adopted, the antenna is not only large in size, but also troublesome to implement. In fact, the slotted structures have strong radiation capability and large radiation loss. Thus, there is a position on the radiation area, after which the guided wave fields is very weak and the contribution to the radiation is small. If we cut off the antenna at this position and make it a single-port leaky-wave structure, the influence on the radiation performance should not be large. In this case, the antenna size has been effectively reduced. The antenna in Fig. S1 is constructed according to this principle. The specific structure is divided into five parts. To do so, the key is the choice for the length of radiation area, i.e. the number of periodic units in the last part. Intuitively, due to the efficient radiation capability of the slits, a relatively sufficient radiation can be achieved with a smaller number of units. In order to verify this idea, the antenna performance with different length radiation area is analyzed here.

For the antenna structure shown in Fig. S1, the number of radiating elements at the tail taken as 6, 8, and 10, respectively. Figure S4 shows the scattering characteristics for these three cases. The almost unchanged port impedance implies that a small number of radiation elements can achieve efficient radiation. Relatively, as the unit number increases, S_11_ value decreases a little. As the number of radiating elements increases, the radiation will be more sufficient. So the reflected wave will decrease and the port matching will be better. In general, 8 radiating elements can meet the truncation requirements.

**
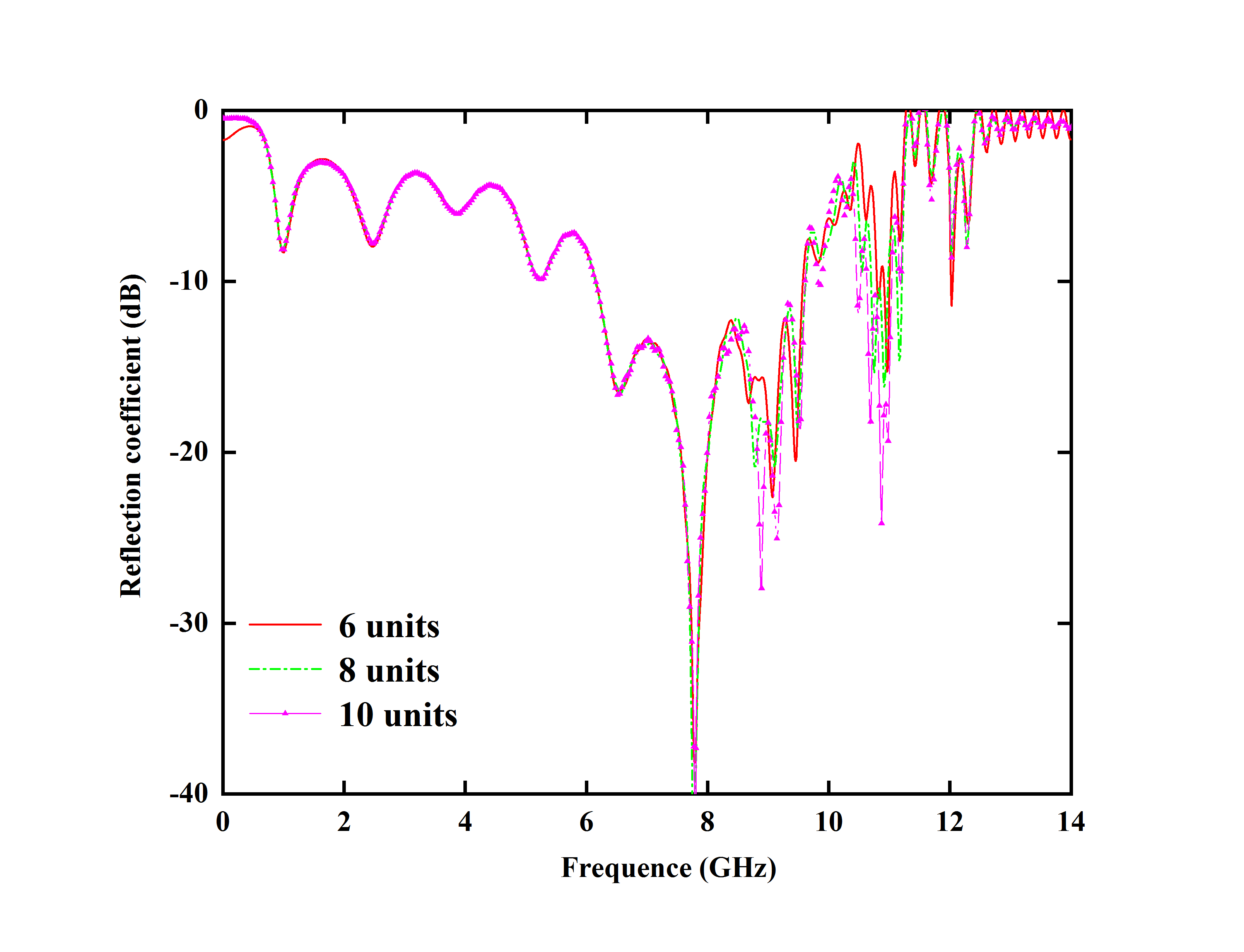
**

**FIG. S4.** The scattering performance of the initial antenna with different number of radiating units.

To further determine the feasibility of this cut-off, Fig. S5 shows the electric field distributions in the *xoz*-cut plane at 7.8 GHz for above three cases. It can be seen that after the CPW port is excited, the TEM mode is first converted to SSPP mode for transmission. The minimum value of the electric fields in the middle part (i.e. the beginning for SSPP waveguide cutting gap) is the division position between the transmission fields and radiation fields. Before that position, the electric field amplitude remained basically unchanged. After that, due to the introduction of radiation gaps, the bound SSPP mode is converted to radiation mode. The electric fields are not only bound to the antenna surface, but also radiate to free space, so the surface field strength continues to decrease.

It can be seen that when the number of radiating elements is 6, the field strength at the tail is still relatively strong. As the number increases to 8, the field strength of tail units is already very weak, and thus their contribution to radiation field is very small. Similar results are obtained when 10 units are selected. The electric field contribution after the 8th unit is negligible, which is consistent with the analysis results of the port scattering characteristics. Therefore, the initial single-port leaky wave antenna shown in Figure S1 uses only 8 radiation units.


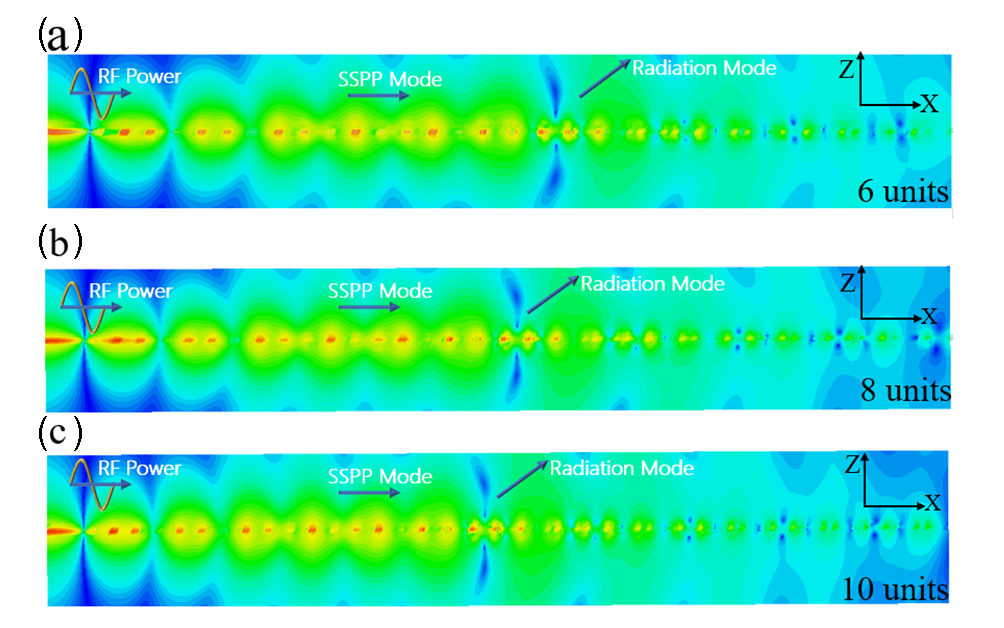


**FIG. S5.** The electric fields in the *xoz* plane with *y* = 5.0 mm (the symmetry plane is at y=0 mm) of the initial leaky wave antenna at 7.8 GHz when the number of radiating elements is (**a**) 6, (**b**) 8, and (**c**) 10. It can be seen that as the number of radiating elements increases from 6 to 10, the electric field intensity at the structure end has decayed to be very weak.

In order to clarify the influence of operating frequency on the radiating element number, Fig. S6 also shows the electric field distributions at 5 frequency points in 6.0–12.0 GHz. In this case, the number of radiating elements is 10. It can also be seen that it is reasonable to choose 8 radiating elements. Because within the band below 10.0 GHz, the electric fields after the 8th element all are already weak. Since the frequency at 10.0 GHz is located in the slow wave zone, there is a strong electric field distribution over the entire structure length. This also shows that the actual application band should be below 10.0 GHz. The cut-off effect at 12.0 GHz is very obvious.


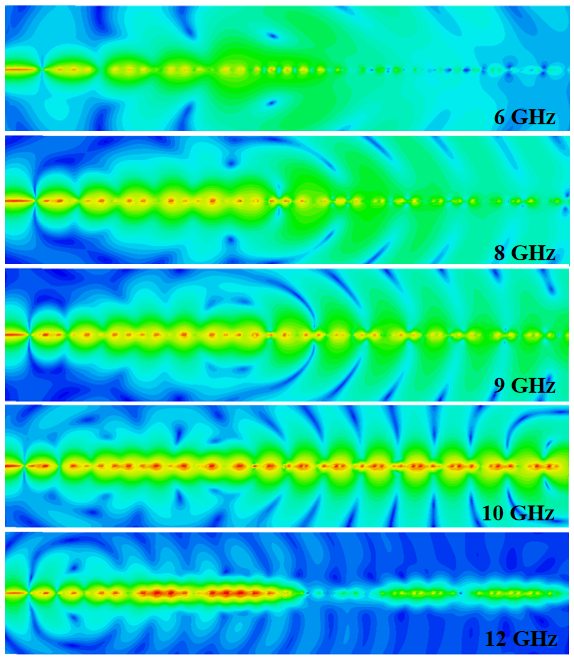


**FIG. S6.** When the number of radiating elements is 10, the surface electric field distributions in the *xoz* plane at 5 frequency points within the range of 6.0–12.0 GHz.

**3. Radiation characteristics of initial single-port leaky wave antenna**

According to the analysis results of the antenna scattering performance and the dispersion characteristics of periodic units, we can conclude that the radiation cut-off frequency of the initial single-port antenna is about 10 GHz. Of course, the radiation characteristics can inversely verify the rationality of the division for the dispersion curve. For this reason, Fig. S7 shows the radiation field characteristics of five frequency points in the band of 7.0–11.0 GHz. It can be seen that the radiation characteristics are consistent with the analysis results of the dispersion curve.

|  | *xoz*-plane pattern | 3D pattern | Gain |
| --- | --- | --- | --- |
| 7.0 GHz | 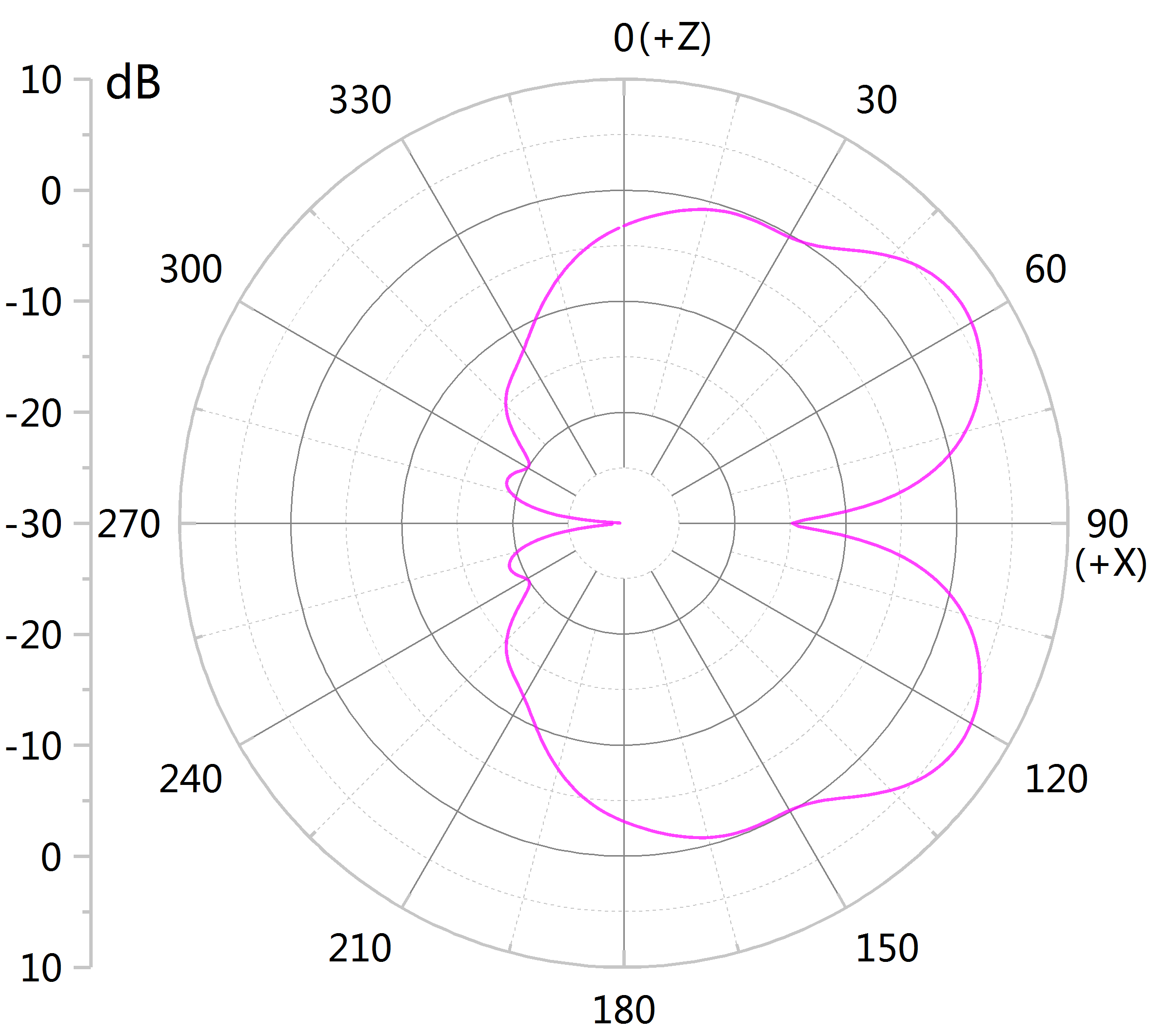 | 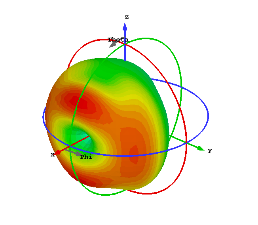 | 6.19 dB |
| 8.0 GHz | 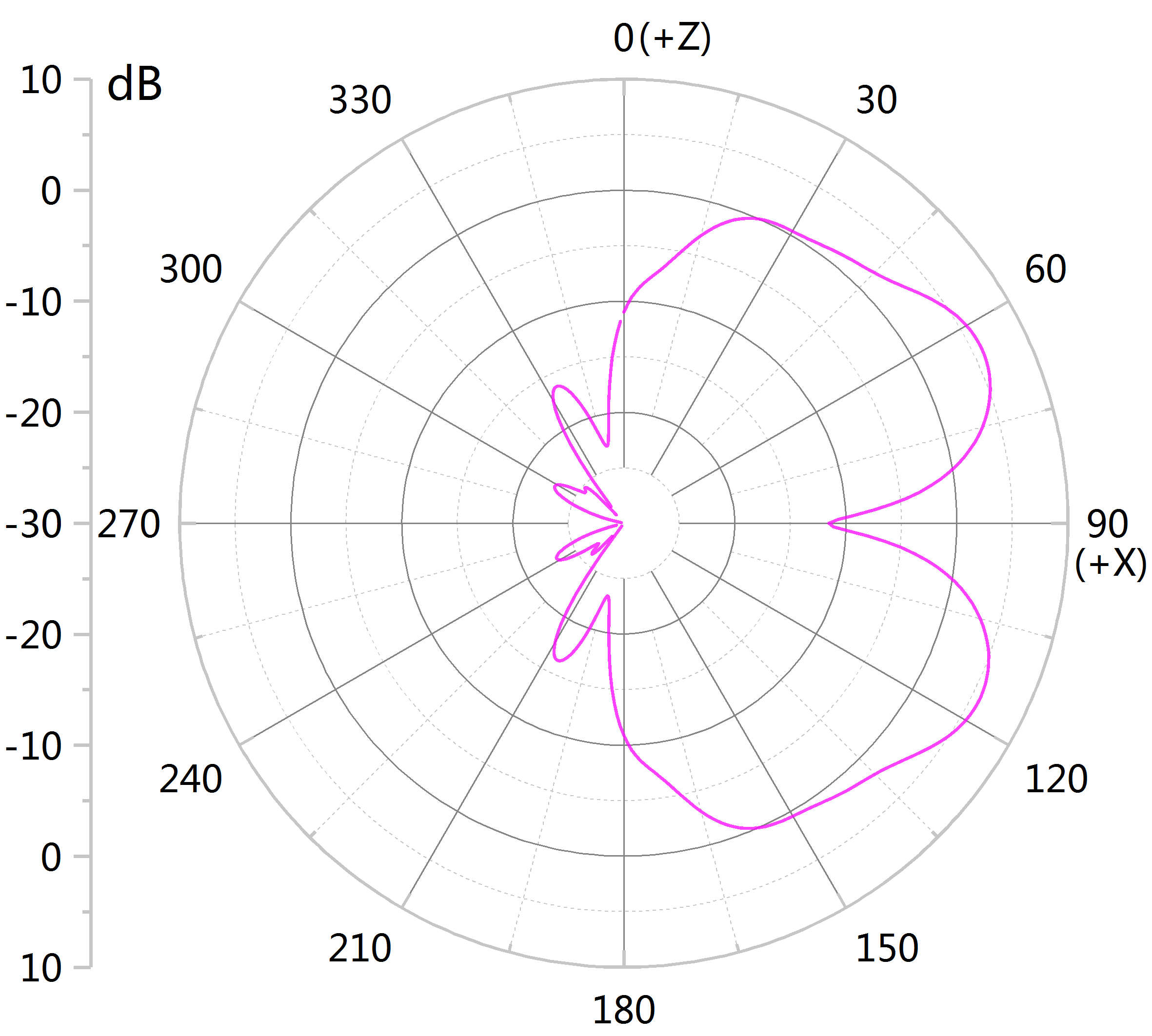 | 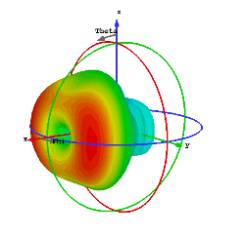 | 8.94 dB |
| 10.0 GHz | 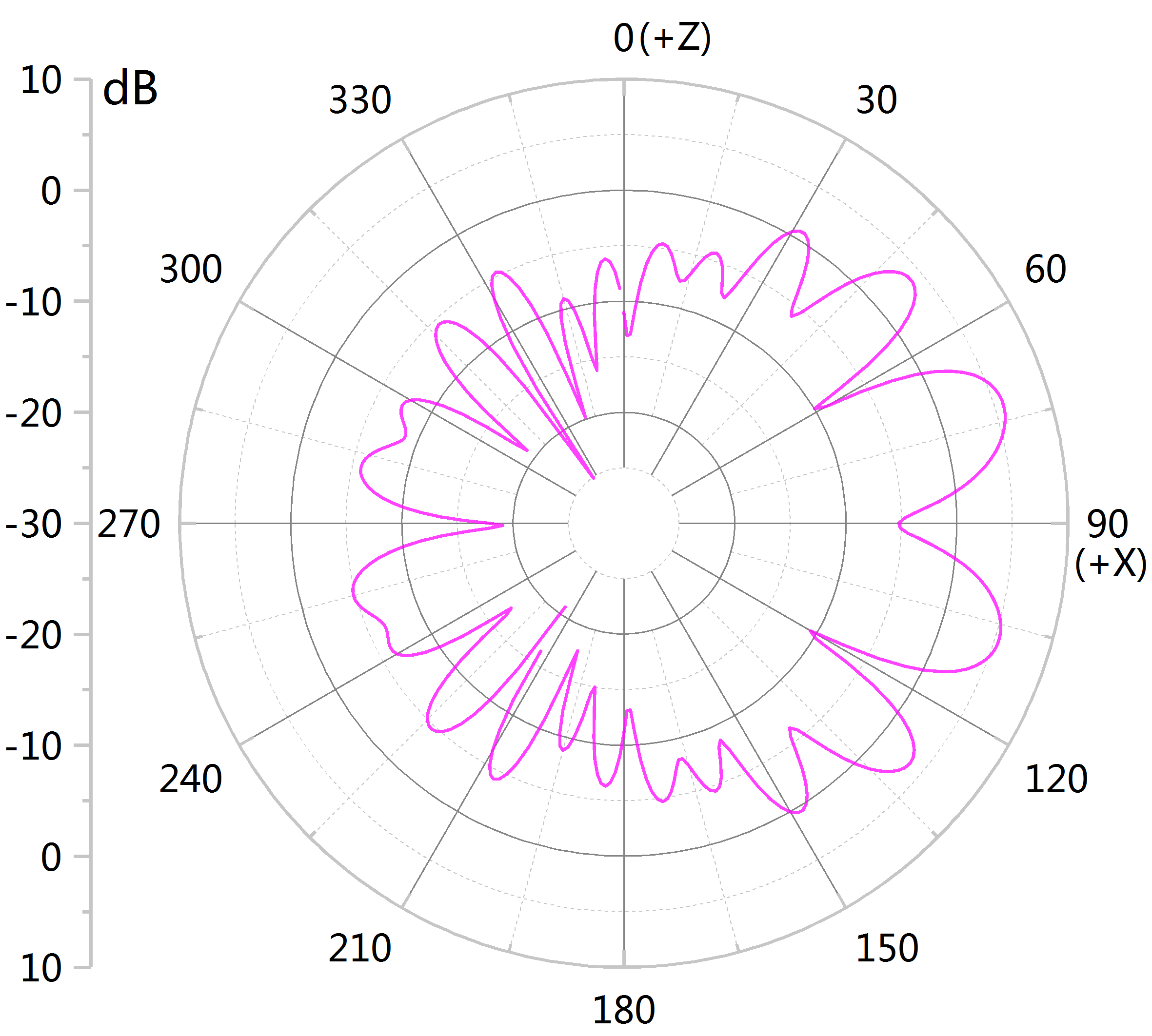 | 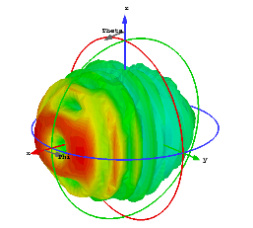 | 6.31 dB |
| 11.0 GHz | 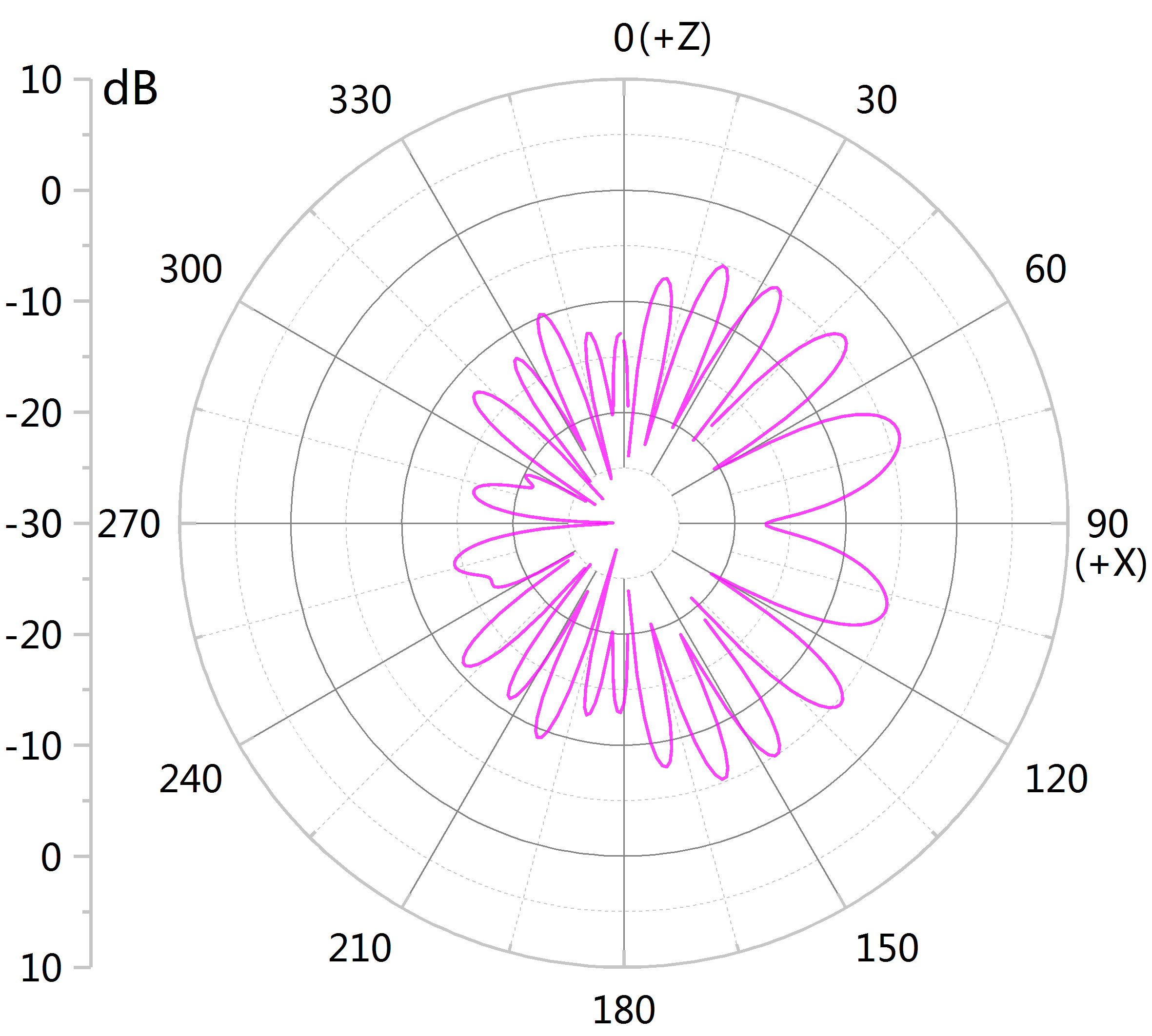 | 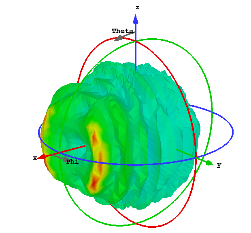 | 1.36 dB |

**FIG. S7.** The simulation radiation patterns and gains for initial single-port antenna. The gain results above 10.0 GHz indicate that the antenna working frequency should be less than 10.0 GHz. On the other hand, as the working frequency is close to 10.0 GHz, the radiation field tends to the antenna axis direction, which is helpful for the realization of radiation on the antenna surface. Therefore, for planar spiral OAM applications, we should take the working frequency band close to 10.0 GHz.

At the same time, in the frequency band less than 10.0 GHz, the maximum radiation direction is closer to the +*x* axis direction as the frequency increases. In other words, taking the vicinity band of the cut-off frequency as the working band can achieve the main radiation near the antenna surface, which also shows that it is feasible to construct a planar spiral orbital angular momentum antenna. At 11.0 GHz, the radiation efficiency of the antenna is already very low, so it should not be used as the operating frequency.
